# Micheliolide Attenuates Lipopolysaccharide-Induced Inflammation by Modulating the mROS/NF-*κ*B/NLRP3 Axis in Renal Tubular Epithelial Cells

**DOI:** 10.1155/2020/3934769

**Published:** 2020-08-17

**Authors:** Xianghong Lei, Shuting Li, Congwei Luo, Yuxian Wang, Yanxia Liu, Zhaozhong Xu, Qianyin Huang, Fangqin Zou, Yihua Chen, Fenfen Peng, Haibo Long

**Affiliations:** ^1^Department of Nephrology, Zhujiang Hospital, Southern Medical University, Guangzhou 510280, China; ^2^Department of Nephrology, The First Affiliated Hospital, Gannan Medical University, Ganzhou, Jiangxi 341000, China; ^3^Department of Gerontology, Zhujiang Hospital, Southern Medical University, Guangzhou 510280, China; ^4^Department of Laboratory Medicine, Zhujiang Hospital, Southern Medical University, Guangzhou 510280, China; ^5^Department of Emergency, Zhujiang Hospital, Southern Medical University, Guangzhou 510280, China

## Abstract

Chronic kidney disease is a common disease closely related to renal tubular inflammation and oxidative stress, and no effective treatment is available. Activation of the nucleotide-binding oligomerization domain-like receptor protein 3 (NLRP3) inflammasome is an important factor in renal inflammation, but the mechanism remains unclear. Micheliolide (MCL), which is derived from parthenolide, is a new compound with antioxidative and anti-inflammatory effects and has multiple roles in tumors and inflammatory diseases. In this study, we investigated the effect of MCL on lipopolysaccharide- (LPS-) induced inflammation in renal tubular cells and the related mechanism. We found that MCL significantly suppressed the LPS-induced NF-*κ*B signaling and inflammatory expression of cytokines, such as tumor necrosis factor-*α* and monocyte chemoattractant protein-1 in a rat renal proximal tubular cell line (NRK-52E). MCL also prevented LPS- and adenosine triphosphate-induced NLRP3 inflammasome activation in vitro, as evidenced by the inhibition of NLRP3 expression, caspase-1 cleavage, and interleukin-1*β* and interleukin-18 maturation and secretion. Additionally, MCL inhibited the reduction of mitochondrial membrane potential and decreases the release of reactive oxygen species (ROS). Moreover, MCL can prevent NLRP3 inflammasome activation induced by rotenone, a well-known mitochondrial ROS (mROS) agonist, indicating that the mechanism of MCL's anti-inflammatory effect may be closely related to the mROS. In conclusion, our study indicates that MCL can inhibit LPS-induced renal inflammation through suppressing the mROS/NF-*κ*B/NLRP3 axis in tubular epithelial cells.

## 1. Introduction

Tubular inflammation plays a central role in the loss of renal function in chronic kidney disease (CKD) [1, 2]. Renal tubules are the main component of the kidney and are vulnerable to various injuries, such as hypoxia, proteinuria, toxins, metabolic disorders, and aging [2]. Proinflammatory cytokines such as interleukin- (IL-) 1*β*, IL-18, tumor necrosis factor-*α* (TNF-*α*), and chemokines, including monocyte chemoattractant protein-1 (MCP-1), and reactive oxygen species (ROS) are involved in the occurrence and progression of tubular inflammation [2–4]. Mounting evidence shows that the nucleotide-binding oligomerization domain-like receptor protein 3 (NLRP3) inflammasome promotes renal inflammation and contributes to CKD [5].

The NLRP3 inflammasome is a multiprotein complex composed of NLRP3, the adaptor protein apoptosis-associated speck-like protein, and effector cysteine protease caspase-1 [6]. NLRP3 recognizes pathogen-associated molecular patterns (PAMPs), such as lipopolysaccharide (LPS) or peptidoglycan, and damage-associated molecular patterns from cells or tissues, such as adenosine triphosphate (ATP). Under the action of agonists, such as bacteria [7], viruses [8], ROS [9], monosodium urate [10], and albumin [11], NLRP3 recruits the adaptor protein apoptosis-associated speck-like protein and caspase-1 to form the macromolecular inflammasome complex. Studies have shown that mitochondrial reactive oxygen species (mROS), mainly derived from mitochondrial oxidative phosphorylation electron leakage, are essential for the activation of the NLRP3 inflammasome [6]. The activated NLRP3 inflammasome cleaves procaspase-1 into enzymatically active caspase-1, which promotes the maturation and secretion of IL-1*β* and IL-18 and results in an inflammatory response [12]. The NLRP3 inflammasome is activated in various kidney diseases, including obstructive nephropathy, diabetic kidney disease, lupus nephritis, IgA nephropathy, minimal change disease, membranous nephropathy, focal segmental glomerulosclerosis, and crescentic glomerulonephritis [5, 13, 14]. Gu et al. found that NLRP3 inflammasome activation was also involved in hyperuricemia-induced renal inflammation, albuminuria, and fibrosis [15, 16]. Therefore, pharmacological inhibitors of the NLRP3 inflammasome have a potential therapeutic value for treating tubular inflammation.

**Figure 1:**
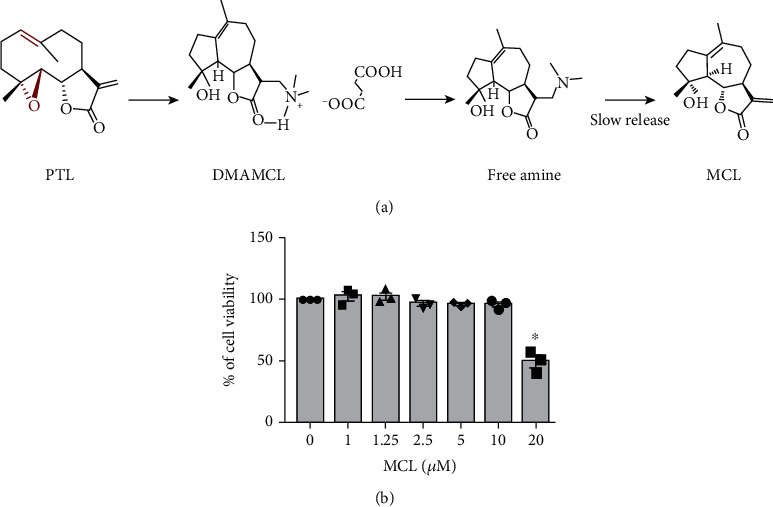
Molecular structure of MCL and its cytotoxicity. (a) Schematic diagram of DMAMCL synthesis from PTL and the mechanism of sustainable release of MCL by DMAMCL under neutral conditions. (b) The MTT reagent was used to assess the cytotoxicity of different concentrations (0, 1, 1.25, 2.5, 5, 10, and 20 *μ*M) of MCL in NRK-52E cells after 48 h of exposure. ^∗^*P* < 0.05 versus normal controls.

As a novel guaianolide sesquiterpene lactone semisynthesized from the well-known NF-*κ*B inhibitor parthenolide (PTL), micheliolide (MCL, Figure 1(a)) exhibits higher stability, lower toxicity, and a longer half-life compared to PTL. Moreover, MCL has been demonstrated to have anti-inflammatory [17, 18], antifibrotic [19, 20], and anticancer [21, 22] effects. Dimethylaminomicheliolide (DMAMCL, i.e., ACT001), the prodrug of MCL, has better water solubility and exerts its therapeutic effects by releasing MCL slowly but consistently in the plasma and in tissues [23]. Most notably, DMAMCL is currently regarded as a potential drug since it has been approved for clinical trials to treat glioblastoma multiforme in Australia (trial ID: ACTRN12616000228482) and China (trial ID: CTR20171274). However, the role of MCL in the activation of the NLRP3 inflammasome in renal tubular inflammation and the related mechanism remain unclear. In this study, we investigated the protective effect of MCL on LPS-induced inflammation in renal tubular epithelial cells and further investigated the potential mechanism of MCL in renal inflammation.

## 2. Materials and Methods

### 2.1. Cell Culture and Treatments

A rat renal proximal tubular cell line (NRK-52E) was kindly provided by Xiao-li Nie, a professor of School of Traditional Chinese Medicine, Southern Medical University. The cells were cultured at 37°C with 5% CO_2_ in DMEM/basic medium (Gibco BRL, USA) supplemented with 10% fetal bovine serum (Gibco BRL, USA) and 1% penicillin/streptomycin (Gibco BRL, USA). To investigate LPS-induced renal inflammation in NRK-52E cells, cells were stimulated with 5 *μ*g/mL LPS (L4391, Sigma) for 3 h and total protein or RNA was extracted. Besides, to investigate the levels of inflammatory cytokine secretion in the supernatant through an enzyme-linked immunosorbent assay experiment, cells were treated with 5 *μ*g/mL LPS for 24 h. To investigate the anti-inflammatory effect of MCL in vitro, cells were pretreated with different concentrations of MCL (1.25 *μ*M, 2.5 *μ*M, and 5 *μ*M) (Accendatech Co., Ltd., Tianjin, China) for 48 h and then incubated with LPS. To induce NLRP3 inflammasome activation, cells were incubated with LPS and then with 3 mM ATP (S1985, Selleck, USA) for 1 h before the cells were extracted. In some experiments, cells were treated with 1 *μ*M mitoquinone (MitoQ) (MedChemExpress) for 24 h or 1 *μ*M rotenone (MedChemExpress) for 24 h.

### 2.2. Cytotoxicity Assay

An MTT assay was used to detect the cytotoxicity of MCL towards NRK-52E cells. Upon reaching 60%-70% confluence, cells were incubated with different concentrations of MCL (0 *μ*M, 1 *μ*M, 1.25 *μ*M, 2.5 *μ*M, 5 *μ*M, 10 *μ*M, and 20 *μ*M) for 48 h. Then, 20 *μ*L per well of MTT (5 mg/mL, Sigma-Aldrich, St. Louis, MO, USA) was added for 4 h in the cell culture incubator. After the culture supernatant was aspirated from each well, 150 *μ*L per well of dimethyl sulfoxide was added and incubated for 10 min at room temperature. The absorbance was measured with a microplate reader at a wavelength of 490 nm.

### 2.3. Western Blotting

Whole-cell lysates of NRK-52E cells were prepared using NP40 cell lysis buffer (Beyotime Institute of Biotechnology), and protein concentrations were quantified using a protein assay kit (Thermo Fisher Scientific, Waltham, MA, USA). Cytoplasmic and nuclear proteins were extracted with the Nuclear/Cytoplasmic Protein Extraction Kit (ComWin Biotech, Beijing, China). Protein samples were separated by electrophoresis on 12% SDS-PAGE and transferred to PVDF membranes (Millipore). The membranes were blocked with 5% nonfat dry milk in TBST for 1 h at room temperature and incubated overnight at 4°C with the following primary antibodies: anti-NLRP3 (1 : 500, BIOSS), anti-MCP-1 (1 : 1000, BOSTER), anti-TNF-*α* (1 : 1,000, BIOSS), anti-IL-1*β* (1 : 500, BIOSS), anti-CASP1 (P10, BOSTER), anti-IL-18 (1 : 500, BIOSS), anti-p65 (1 : 1000, Cell Signaling Technologies), anti-PCNA (1 : 500, Wanleibio), anti-*β*-actin (1 : 5000, EarthOx), and *anti-α*-tubulin (1 : 5000, Beijing Ray Antibody Biotech). The washed membranes were then incubated with matched HRP-conjugated rabbit or mouse secondary antibodies (1 : 5000, EarthOx) for 1 h at room temperature and visualized with ECL plus western blotting detection reagents (Millipore). The relative expression of each protein was normalized to the internal control protein *β*-actin or *α*-tubulin. Protein band densities were quantified with Photoshop CS5 software (Adobe System Inc.).

### 2.4. Quantitative Real-Time PCR Analysis (qPCR)

Total RNAs were extracted from the cells using the TRIzol reagent (TransGen Biotech, Beijing, China). A total of 1 *μ*g RNA was used to synthesize cDNA, which was used as a template for PCR by the use of a SYBR Green Master Mix kit (Takara Biotechnology, Shiga, Japan) according to the manufacturer's protocol. Primers were shown as follows: TNF-*α*: forward primer: 5′-ATGGGCTCCCTCTCATCAGTTCC-3′ and reverse primer: 5′-GCTCCTCCGCTTGGTGGTTTG-3′, MCP-1: forward primer: 5′-CAGCCCAGAAACCAGCCAACTC-3′ and reverse primer: 5′-CAACAGGCCCAGAAGCGTGAC-3′, IL-1*β*: forward primer: CTCACAGCAGCATCTCGACAAGAG and reverse primer: TCCACGGGCAAGACATAGGTAGC, and *β*-actin: forward primer: 5′-GTGACGTTGACATCCGTAAAGA-3′ and reverse primer: 5′-GCCGGACTCATCGTACTCC-3′. To quantify the expression of target genes, *β*-actin was used as an internal reference. The results were calculated by the 2^-*ΔΔ*Ct^ method.

### 2.5. Enzyme-Linked Immunosorbent Assay (ELISA)

The supernatant of cultured cells was collected, and the levels of MCP-1 (CSB-E07429r, Cusabio) and TNF-*α* (KRC3011, Invitrogen) and IL-18 (EK0592, BOSTER) were determined with an ELISA kit according to the manufacturer's protocols. The photometric measurements were performed at 450 nm using a microplate reader.

### 2.6. Determination of Intracellular ROS by Flow Cytometry

The level of intracellular ROS was measured with a ROS Assay Kit (S0033, Beyotime Institute of Biotechnology). The oxidant-sensitive fluorescent probe 2′,7′-dichlorofluorescein diacetate (DCFH-DA) diffuses easily into cells and is deacetylated to form nonfluorescent 2′,7′-dichlorofluorescein (DCFH), which reacts with ROS to form the highly fluorescent 2′,7′-dichlorofluorescein (DCF). After treatment with LPS and different concentrations of MCL, the cells were trypsinized and collected in ice-cold PBS. Cells were then incubated with 10 *μ*M DCFH-DA at 37°C for 20 min. Cells were collected by centrifugation, washed, and resuspended in PBS. The intracellular ROS, as indicated by DCF fluorescence intensity, was measured with a flow cytometer.

### 2.7. Measurement Analysis of Mitochondrial Membrane Potential (MMP)

The MMP of NRK-52E cells was measured using an MMP assay kit with JC-1 (5,5′,6′,6-tetrachloro-1,1′,3,3′-tetraethylbenzimidazolcarbocyanine iodide; C2006; Beyotime Institute of Biotechnology), a cationic stain accumulating on mitochondrial membrane yielding red fluorescence in healthy mitochondria whereas it displayed green fluorescence in depolarized or damaged mitochondria. The kit provides carbonyl cyanide 3-chlorophenylhydrazone (CCCP) as a positive control for inducing a decrease in MMP. The experimental operation was carried out according to the manufacturer's instructions. Briefly, cells were pretreated with 5 *μ*M MCL for 48 h and incubated with LPS for 3 h. Then, the cells were incubated with JC-1 dye at 37°C for 20 min. After aspirating the supernatant and washing, the cell culture medium was added, and the fluorescence was measured using a fluorescence microscope (Leica). The red/green fluorescence intensity ratio was used to express the change in mitochondrial membrane potential.

### 2.8. Statistical Analysis

All the data are presented as the mean ± SEM of at least three independent experiments. Statistical analyses were performed with one-way ANOVA using SPSS for Windows version 20 (SPSS, Chicago, IL, USA). *P* < 0.05 was considered significant.

## 3. Results

### 3.1. MCL Alleviates the LPS-Induced NK-*κ*B-Dependent Inflammatory Response in NRK-52E Cells

We initially tested the cytotoxicity of MCL using an MTT assay. The results showed that low concentrations (1-10 *μ*M) of MCL had no significant effect on cell viability after 48 h of incubation. However, high concentrations (20 *μ*M) of MCL significantly reduced the survival rate of NRK-52E cells (Figure 1(b)). Therefore, MCL concentrations of 1.25, 2.5, and 5 *μ*M were used in our subsequent experiments.

**Figure 2:**
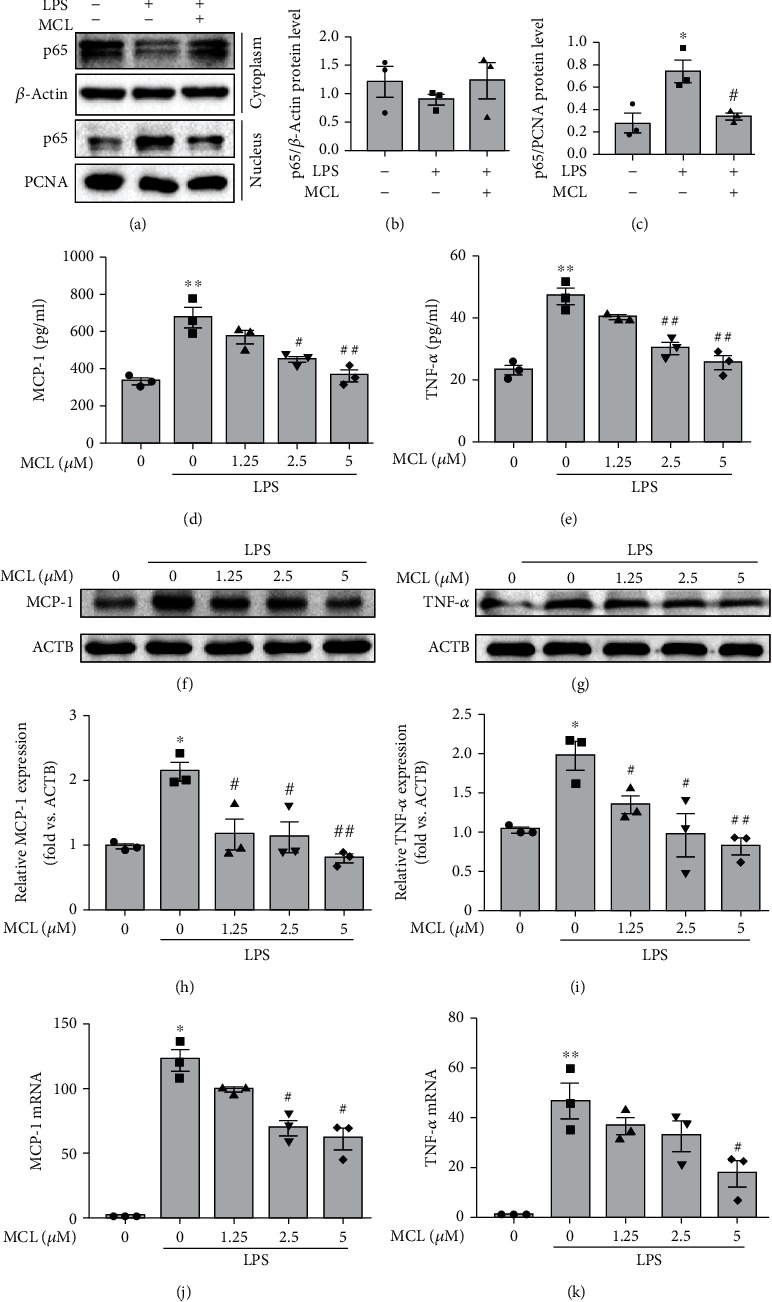
MCL alleviates the LPS-induced NK-*κ*B-dependent inflammatory response in NRK-52E cells. (a) NRK-52E cells were treated with LPS with or without MCL (5 *μ*M). Cytoplasmic and nuclear proteins were extracted, and p65 protein expression was detected by western blotting. (b) Cytoplasmic p65 expression relative to *β*-actin was quantified. (c) Nuclear p65 expression relative to PCNA was quantified. Data are presented as the mean ± SEM. ^∗^*P* < 0.05 versus normal controls; ^#^*P* < 0.05 versus the LPS stimulation group. (d, e) ELISA analysis of (d) MCP-1 and (e) TNF-*α* expression in each group. (f, g) Western blot analysis of (f) MCP-1 and (g) TNF-*α* expression. (h, i) The relative expression levels of the indicated proteins which were normalized to *β*-actin (ACTB) expression. (j, k) Real-time PCR analysis of (j) MCP-1 and (k) TNF-*α* expression in renal tubular epithelial cells. Data are presented as the mean ± SEM. ^∗^*P* < 0.05, ^∗∗^*P* < 0.01 versus normal controls; ^#^*P* < 0.05, ^##^*P* < 0.01 versus the LPS stimulation group.

As shown in Figures 2(a)–2(c), MCL blunted LPS-induced nuclear translocation of p65, indicating that MCL inhibits LPS-induced activation of the NF-*κ*B pathway. To investigate the role of MCL in NF-*κ*B-dependent inflammatory responses, we examined the level of inflammatory cytokines in NRK-52E cells induced by LPS with or without MCL intervention. The ELISA results showed that compared to the control group, LPS exposure resulted in a significant increase in the levels of the cellular inflammatory factors such as MCP-1 and TNF-*α*, suggesting that LPS induces an inflammatory response in rat renal tubular cells. However, MCL pretreatment inhibited LPS-induced inflammatory factor expression in a concentration-dependent manner (Figures 2(d) and 2(e)). We obtained similar results through immunoblotting and qPCR experiments (Figures 2(f)–2(k)). Taken together, these data indicate that MCL alleviates the LPS-induced NK-*κ*B-dependent inflammatory response in rat renal tubular cells.

### 3.2. MCL Inhibits LPS+ATP-Induced Activation of the NLRP3 Inflammasome in NRK-52E Cells

**Figure 3:**
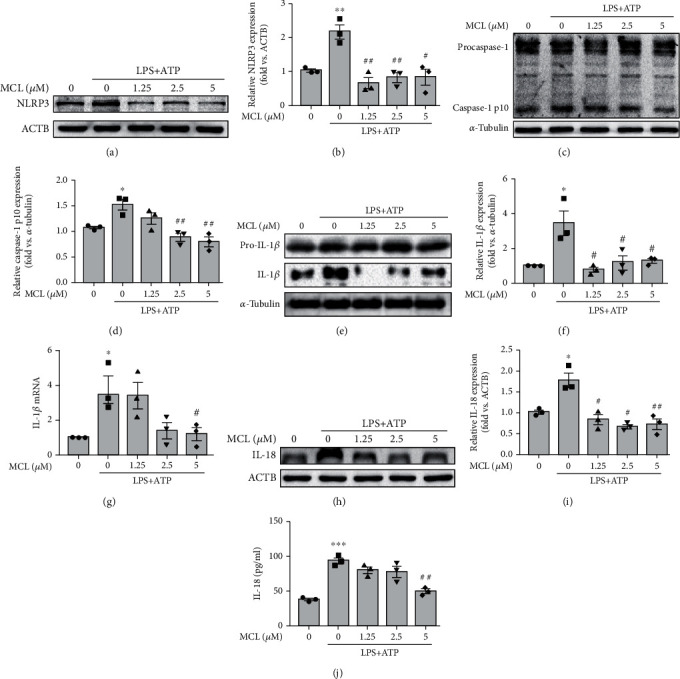
MCL inhibits LPS+ATP-induced activation of the NLRP3 inflammasome in NRK-52E cells. (a, b) Western blot analysis of the NLRP3 expression and its relative expression levels normalized to *β*-actin (ACTB). (c, d) Western blot analysis of the caspase-1 p10 expression and its relative expression levels normalized to *α*-tubulin. (e, f) Western blot analysis of the IL-1*β* expression and its relative expression levels normalized to *α*-tubulin. (g) Real-time PCR analysis of IL-1*β* expression in renal tubular epithelial cells. (h, i) Western blot analysis of the IL-18 expression and its relative expression levels normalized to ACTB. (j) ELISA analysis of IL-18 expression in each group. Data are presented as the mean ± SEM. ^∗^*P* < 0.05, ^∗∗^*P* < 0.01 versus normal controls; ^#^*P* < 0.05, ^##^*P* < 0.01 versus the LPS+ATP stimulation group.

As known, LPS plus ATP can induce NLRP3 inflammasome activation [24]. In our study, the expression levels of NLRP3, caspase-1 p10, IL-1*β*, and IL-18 in the LPS+ATP stimulation group were significantly upregulated compared with those in the control group, indicating that the NLRP3 inflammasome was activated (Figure 3). After MCL pretreatment at different concentrations (1.25 *μ*M, 2.5 *μ*M, and 5 *μ*M), the expression levels of NLRP3 (Figures 3(a) and 3(b)), IL-1*β* (Figures 3(e) and 3(f)), caspase-1 p10 (Figures 3(c) and 3(d)), and IL-18 (Figures 3(h) and 3(i)) were significantly reduced compared with those in the LPS+ATP stimulation group. Besides, qPCR analysis revealed that MCL reduced IL-1*β* mRNA expression upregulated by LPS+ATP (Figure 3(g)). The ELISA results showed that the IL-18 level was decreased after MCL pretreatment compared with that in the LPS+ATP stimulation group (Figure 3(j)). The above results indicate that MCL treatment inhibits the activation of the NLRP3 inflammasome.

### 3.3. MCL Inhibits the LPS-Induced Reduction in MMP in NRK-52E Cells

**Figure 4:**
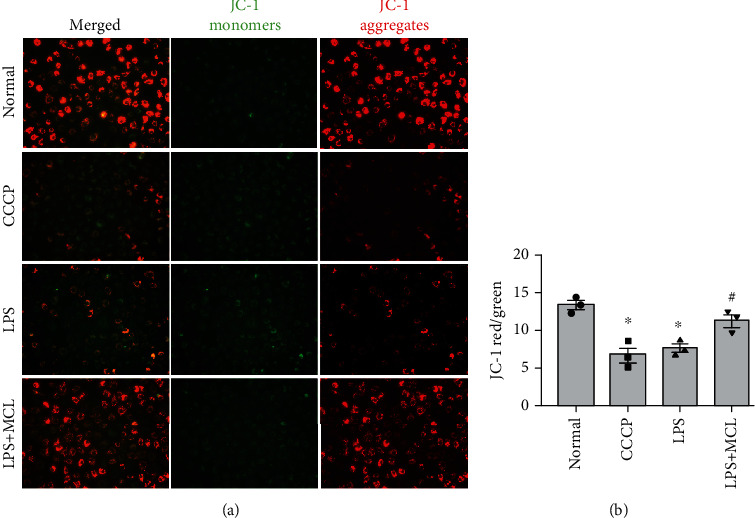
MCL inhibits the LPS-induced MMP reduction. (a) MMP in NRK-52E cells was analyzed by JC-1 staining (200x). (b) Fluorescence analysis of the MMP of each group. ^∗^*P* < 0.05 versus normal controls; ^#^*P* < 0.05 versus the LPS stimulation group. CCCP: the kit provides a positive control for inducing a decrease in MMP.

Mitochondria are the main sites where cells make ATP. Normal mitochondrial membrane potential is a prerequisite for maintaining mitochondrial oxidative phosphorylation and ATP production, which is crucial for maintaining mitochondrial function [25]. In our study, fluorescence detection showed that in cells treated with the mitochondrial electron transport chain inhibitor CCCP, the red-green fluorescence ratio decreased significantly, indicating that the MMP was sufficiently decreased (Figure 4). Besides, cells stimulated with LPS also showed a decrease in the MMP (Figure 4). MCL pretreatment significantly restored membrane potential which was disrupted with LPS (Figure 4), suggesting the proposed mitoprotective property of this new compound.

### 3.4. MCL Inhibits NLRP3 Inflammasome Activation through Suppressing the mROS in NRK-52E Cells

**Figure 5:**
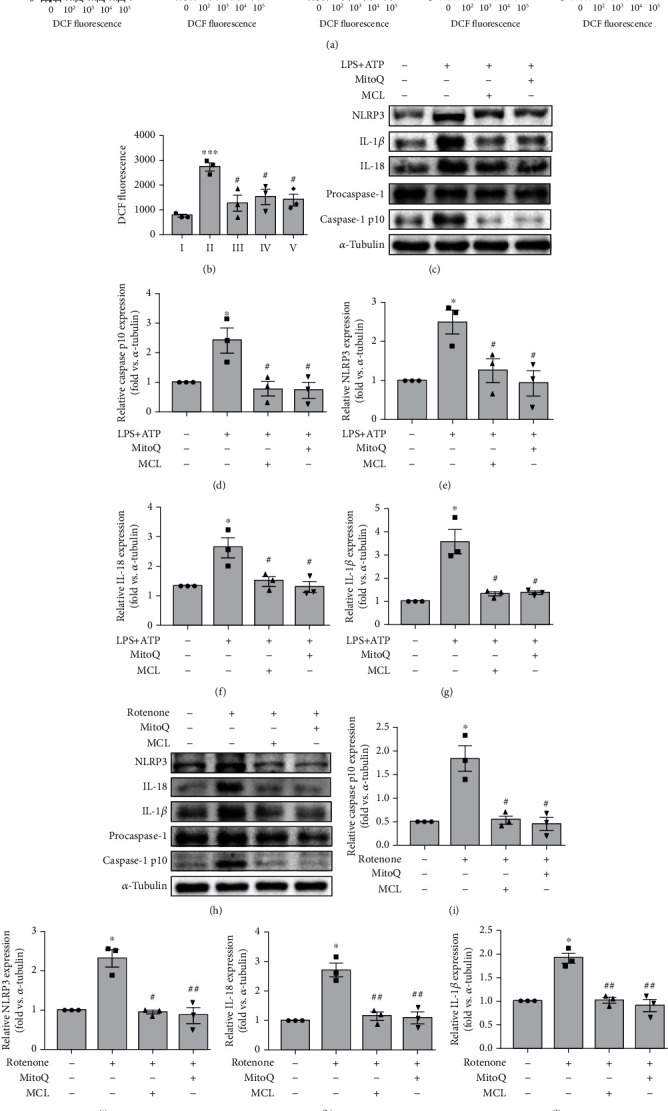
MCL inhibits NLRP3 inflammasome activation through suppressing the mROS in NRK-52E cells. (a) Flow cytometry analysis of the release of ROS in each group. (b) Comparative analysis of the amount of ROS released in each group. ^∗∗∗^*P* < 0.001 versus normal controls; ^#^*P* < 0.05 versus the LPS stimulation group. (c) Western blot analysis of NLRP3, IL-1*β*, IL-18, and caspase-1 p10 expression in renal tubular epithelial cells treated with MitoQ (1 *μ*M) or MCL (5 *μ*M) and stimulated with LPS+ATP. (d–g) The relative expression levels of the indicated proteins normalized to *α*-tubulin expression. Data are presented as the mean ± SEM. ^∗^*P* < 0.05 versus normal controls; ^#^*P* < 0.05 versus the LPS+ATP stimulation group. (h) Western blot analysis of NLRP3, IL-1*β*, IL-18, and caspase-1 p10 expression in renal tubular epithelial cells treated with MitoQ or MCL (5 *μ*M) and stimulated with rotenone. (i–l) The relative expression levels of the indicated proteins normalized to *α*-tubulin expression. Data are presented as the mean ± SEM. ^∗^*P* < 0.05 versus normal controls; ^#^*P* < 0.05, ^##^*P* < 0.01 versus the rotenone stimulation group.

To assess the effect of MCL on intracellular ROS production, we measured ROS level using DCFH-DA in NRK-52E cells. The flow cytometry results showed that the release of ROS in the LPS-stimulated group was significantly higher than that in the control group, and the release of ROS was significantly reduced after MCL intervention (Figures 5(a) and 5(b)). Furthermore, we used the mROS inhibitor MitoQ in vitro experiments. The immunoblotting results showed that compared with the LPS+ATP groups without MitoQ pretreatment, pretreatment with MitoQ significantly inhibited the activation of the NLRP3 inflammasome, as evidenced by the decreased expression of NLRP3, IL-1*β*, IL-18, and caspase-1 p10 (Figures 5(c)–5(g)). Rotenone is a mitochondrial complex I inhibitor which can promote mROS production [26]. In our study, we found that rotenone induced NLRP3 inflammasome activation, whereas MCL or MitoQ inhibited rotenone-induced NLRP3 inflammasome activation (Figures 5(h)–5(l)), suggesting that MCL inhibits the activation of the NLRP3 inflammasome by reducing the release of mROS.

## 4. Discussion

Chronic inflammation and oxidative stress play an important role in the pathogenesis and progression of CKD. NLRP3 inflammasome activation is involved in several inflammatory diseases. MCL has multiple roles in tumors and inflammatory diseases. We previously found that MCL reduces renal inflammation by blocking the NF-*κ*B pathway [17, 18]. Here, in this study, our data showed that MCL can inhibit the activation of the NF-*κ*B pathway and NLRP3 inflammasome by inhibiting the release of mROS, thereby ameliorating tubular inflammation. These data suggest that MCL may represent a promising therapeutic strategy for renal inflammation in CKD.

Renal inflammatory injury induces inflammatory cell infiltration via modulation of chemokines [27]. MCP-1 is the most important chemokine involved in regulating monocytes in inflammatory diseases and is a key player in the pathogenesis of inflammatory nephropathy [28, 29]. The main characteristic of MCP-1 is its chemoattractant effect on monocytes/macrophages [30]. These inflammatory cells in turn secrete inflammatory factors and chemokines that promote inflammation and fibrosis. A variety of cells of normal kidney tissue, such as glomerular endothelial cells, mesangial cells, and tubular cells, secrete trace amounts of MCP-1. When renal tissue is stimulated, the expression of MCP-1 is significantly higher than that in unstimulated kidney tissue, and MCP-1 expression positively correlates with the degree of renal damage [30]. TNF-*α* and IL-1*β* are important inflammatory factors that can promote the secretion of MCP-1. In this study, LPS stimulated the expression of the inflammatory factors MCP-1 and TNF-*α* in NRK-52E cells. This finding confirms that LPS stimulation can induce tubular inflammation. Furthermore, MCL significantly inhibited LPS-induced activation of NF-*κ*B and the expression of these inflammatory factors driven by NF-*κ*B. These data indicate that MCL can significantly inhibit the tubular inflammatory response.

The inflammasome is a multiprotein complex assembled by intracytoplasmic pattern recognition receptors. Inflammasomes recognize PAMPs or host-derived risk signaling molecules and recruit and activate the proinflammatory protein caspase-1. Caspase-1 plays a decisive role in pyroptosis. The activation of caspase-1 results in intracellular bacterial clearance in vivo and induces pyroptosis as an efficient mechanism of bacterial clearance by the innate immune system [31]. Activated caspase-1 cleaves precursors of IL-1*β* and IL-18, producing the corresponding mature cytokines [32]. IL-1*β* and IL-18 are members of the IL-1 superfamily and are important proinflammatory factors in cells. IL-1*β* is mainly secreted by mononuclear macrophages, which can further trigger an inflammatory response and play a central role in local and systemic inflammatory responses. IL-18 is mainly produced by activated macrophages, which stimulate Th1 cells to secrete granulocyte-macrophage colony-stimulating factor, IFN-*γ*, and IL-2 and promote the proliferation of Th1 cells. IL-1*β* and IL-18 can increase the expression of *α*-SMA protein in renal tubular epithelial cells, promote the transdifferentiation of renal tubular epithelial cells into myofibroblasts, and promote renal interstitial fibrosis [33, 34]. Currently, the NLRP3 inflammasome is the most widely studied inflammasome. NLRP3 inflammasome activation can be divided into two necessary phases. The first stage is induced by NF-*κ*B pathway activation, initiating IL-1*β*, IL-18, and NLRP3 transcription and translation. The second stage is related to the assembly of the NLRP3 inflammasome [35, 36]. The NLRP3 inflammasome contributes to a wide range of acute and chronic kidney diseases via mechanisms that regulate inflammation, pyroptosis, apoptosis, and fibrosis [37]. Therefore, inhibition of NLRP3 inflammasome activation is a new strategy for the treatment of CKD. LPS is an endotoxin that has been shown to promote activation of the NLRP3 inflammasome and increase the expression of proinflammatory cytokines such as IL-1*β* and IL-18 [38, 39], which is consistent with our experimental results. Our results show that MCL significantly inhibits LPS-induced activation of the NLRP3 inflammasome in NRK-52E cells and reduces the expression of downstream factors such as caspase-1, IL-1*β*, and IL-18, suggesting that MCL attenuates LPS-induced inflammation by inhibiting the activation of the NF-*κ*B and NLRP3 inflammasome in NRK-52E cells.

Mitochondria are the major sites of cellular aerobic respiration and play a key role in regulating pattern recognition receptor signaling pathways [39], including the regulation of NF-*κ*B and NLRP3 inflammasome activation. Mitochondria are the main source of ROS. External stimuli promote the mitochondrial production of ROS during signal transduction, which in turn stimulates signaling pathways and participates in cellular signaling processes that alert the immune system [40–42]. Studies found that mROS production mediates LPS-induced NF-*κ*B activation [43] and NLRP3 activation [44, 45]. Lysosomal membrane permeabilization caused by the release of mROS is essential for NLRP3 activation. When mitochondrial function declines, ROS production increases, causing damage to tissues and organs. In addition, mitochondria are also the main targets of ROS damage. The accumulation of ROS stimulates the continuous opening of mitochondrial membrane pores, causing a decrease in MMP, respiratory chain abnormalities, and the induction of mitochondrial protein release and cell death [44]. To explore the effect of MCL on mitochondrial function in renal tubular epithelial cells, we examined the levels of ROS and MMP in each group of cells. Our data show that MCL administration significantly inhibits LPS-induced cellular ROS release and MMP reduction and that the mROS inhibitor MitoQ significantly inhibits LPS-induced activation of the NLRP3 inflammasome. MCL or MitoQ inhibited rotenone-induced NLRP3 inflammasome activation, suggesting that MCL inhibits the activation of the NLRP3 inflammasome by reducing the release of mROS. Hence, we speculate that MCL alleviates the LPS-induced inflammatory response in rat renal tubular cells through the mROS/NF-*κ*B/NLRP3 pathway.

## 5. Conclusions

**Figure 6:**
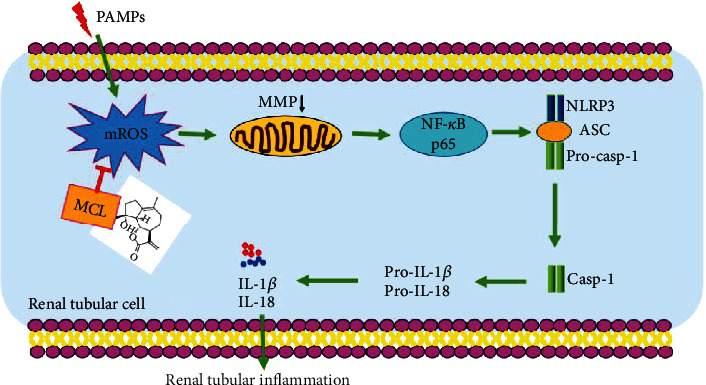
Schematic diagram of the mechanism by which MCL attenuates the inflammatory response of NRK-52E cells. MCL inhibits the activation of the NF-*κ*B pathway and NLRP3 inflammasome by inhibiting the release of mROS, thereby ameliorating tubular inflammation.

In conclusion, this study shows that MCL significantly reduces renal tubular epithelial cell inflammation. Besides, MCL inhibits the activation of the NF-*κ*B and NLRP3 inflammasome by inhibiting the release of mROS, thereby ameliorating the inflammatory response. Our study provides a novel theoretical basis for the use of MCL to treat CKD (Figure 6).

## Data Availability

The data used to support the findings of this study are available on request to the corresponding author.

## Abbreviations

MCL: Micheliolide
NLRP3: Nucleotide-binding oligomerization domain-like receptor protein 3
LPS: Lipopolysaccharide
CKD: Chronic kidney disease
ROS: Reactive oxygen species
ATP: Adenosine triphosphate
mROS: Mitochondrial reactive oxygen species
qPCR: Quantitative real-time PCR analysis
DCFH-DA: 2′,7′-Dichlorofluorescein diacetate
DCFH: 2′,7′-Dichlorofluorescein
DCF: 2′,7′-Dichlorofluorescein
MMP: Mitochondrial membrane potential
CCCP: Carbonyl cyanide 3-chlorophenylhydrazone
MitoQ: Mitoquinone
IL-1*β*: Interleukin-1*β*
IL-18: Interleukin-18
TNF-*α*: Tumor necrosis factor-*α*
MCP-1: Monocyte chemoattractant protein-1.
